# Case Report: Successful management of high-volume paclitaxel extravasation with hyaluronidase and dry warm compresses

**DOI:** 10.3389/fonc.2025.1699608

**Published:** 2025-12-08

**Authors:** Boris Dudík, Maroš Kunderlík, Bela Mriňáková, Zuzana Lukáčová, Vanda Ušáková, Ján Klimas

**Affiliations:** 1 Pharmacy of St. Elisabeth, St. Elisabeth Cancer Institute, Bratislava, Slovakia; 2 Department of Pharmacology and Toxicology, Faculty of Pharmacy, Comenius University Bratislava, Bratislava, Slovakia; 3 1st Department of Oncology, Faculty of Medicine, Comenius University Bratislava and St. Elisabeth Cancer Institute, Bratislava, Slovakia

**Keywords:** paclitaxel, extravasation, vesicants, hyaluronidase, warm compresses

## Abstract

**Background:**

Paclitaxel is a widely used chemotherapeutic agent with known vesicant properties. Extravasation is rare, but it can result in significant local tissue injury. There is no consensus on the optimal management strategy, and recommendations differ significantly.

**Case presentation:**

We report the case of a 63-year-old woman with metastatic cervical cancer who experienced a large-volume paclitaxel extravasation during a peripheral infusion. Management involved immediate subcutaneous administration of hyaluronidase around the affected area, followed by repeated applications of dry warm compresses. The patient reported only mild discomfort during treatment, and the local cutaneous symptoms resolved within days. Despite effective local management, the patient developed persistent paresthesia in the extravasated limb, later diagnosed as sensory neuropathy affecting the ulnar nerve. This was accompanied by lower limb neuropathy, likely related to the systemic paclitaxel exposure. Paclitaxel was discontinued, and the patient continued palliative treatment with carboplatin monotherapy.

**Conclusion:**

Based on our literature search, this appears to be the first documented case of high-volume paclitaxel extravasation successfully managed with both hyaluronidase and dry warmth. The intervention proved to be effective in preventing severe local tissue injury, although it did not mitigate neurotoxic effects. Further research is needed to establish standardized management protocols.

## 1 Introduction

Extravasation is an unintended event that occurs during parenteral drug administration, in which the drug or other infused fluid leaks from the blood vessel into the surrounding tissues. Extravasations most commonly present with pain, a burning or stinging sensation, and edema at the infusion site. In cases involving chemotherapeutic agents, tissue damage of varying intensity and extent may occur, depending on the type and volume of the leaked substance (1).

There has been a debate about whether taxanes should be classified as vesicants, whose extravasation may lead to cellulitis, skin fibrosis and skin necrosis, or as irritants, which typically cause inflammation, pain and irritation but rarely result in serious tissue damage. Barbee et al. classified paclitaxel as a vesicant, based on reports of blistering following extravasation in both animal studies and patient cases (2). The potential for serious skin and tissue damage after paclitaxel extravasation is also noted by the manufacturer in the Summary of Product Characteristics (SmPC) (3).

Reports of extravasation incidents associated with cancer drug therapy are scarce in the literature (4–7). Due to the relative rarity of these events, well-established management protocols are lacking. However, a recent study has demonstrated that, despite the limited number of published reports, extravasation events remain quite common in clinical practice and can lead to severe consequences, particularly in cases involving vesicant agents (8). Current practices and guidelines are often not supported by controlled studies and are instead based on a limited number of case reports or small retrospective cohorts (2). Specifically, recommendations for managing paclitaxel extravasation are unclear and vary considerably. Earlier sources recommended only observation and dry cold compresses to prevent further spread of the extravasate (9). The European Society for Medical Oncology (ESMO) and European Oncology Nursing Society (EONS) guidelines from 2012 recommend a markedly different approach: application of hyaluronidase along with dry warm compresses to promote dispersion and dilution of paclitaxel (10). More recently, Japanese Society of Cancer Nursing (JSCN), Japanese Society of Medical Oncology (JSMO), and Japanese Society of Pharmaceutical Oncology (JASPO) guidelines published in 2024 do not support the use of warm compresses and hyaluronidase following paclitaxel extravasation based on the lack of evidence supporting this approach for taxanes (the available data support this approach exclusively for vinca alkaloid extravasation) (1). They recommend, albeit only weakly, the use of cold compresses as a local therapy to prevent exacerbation or progression of extravasation (1). The latest Oncology Nursing Society/American Society of Clinical Oncology (ONS–ASCO) guidelines conditionally recommend hyaluronidase for taxane (including paclitaxel) extravasation and list warm compresses as appropriate management, noting very low level of evidence (11). In our case, high-volume paclitaxel extravasation was managed with combination of hyaluronidase and dry warmth according to our institutional protocol based on 2012 ESMO–EONS guidelines (10).

## 2 Case description

### 2.1 Initial phase

**Figure 1:**
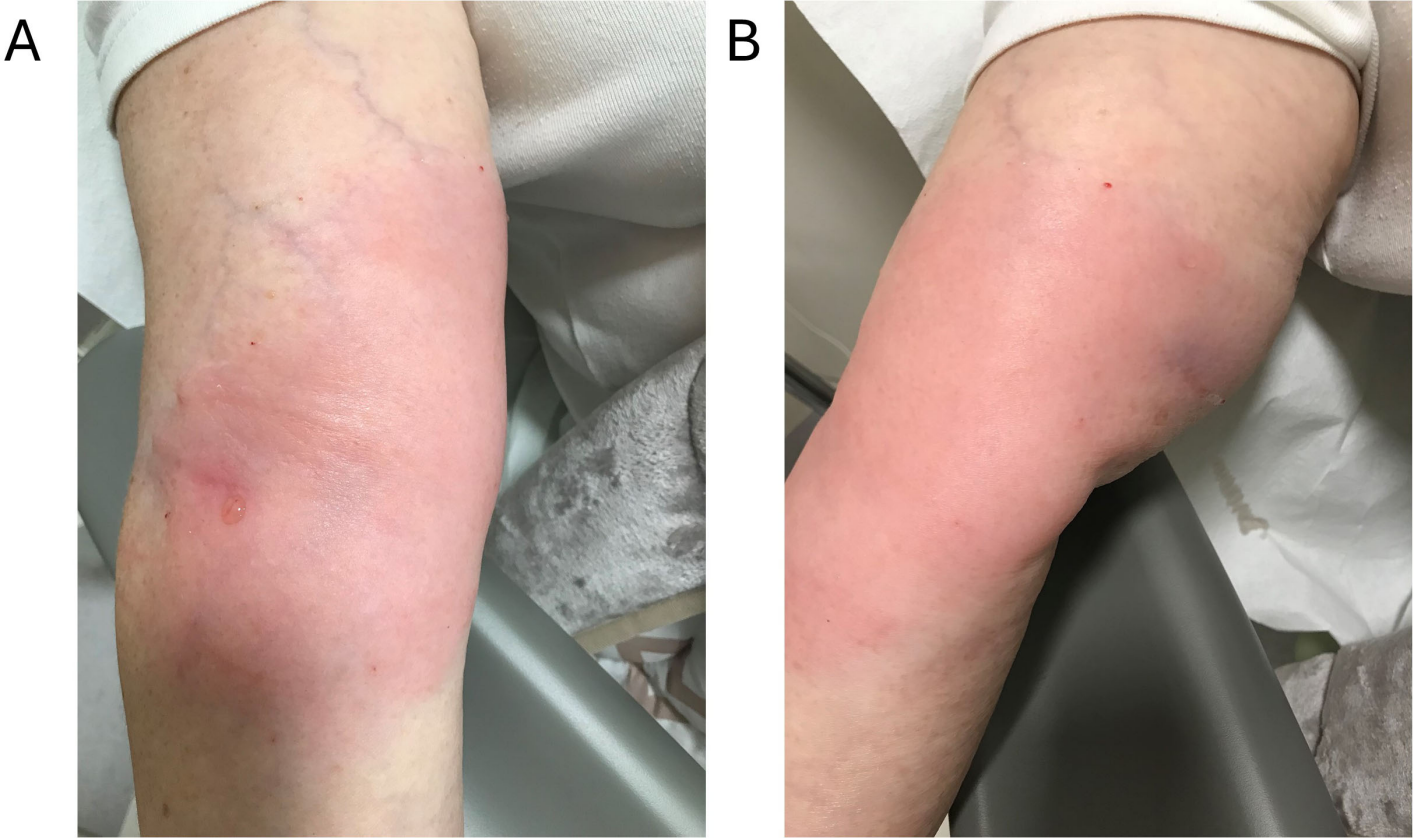
Paclitaxel extravasation. September 9^th^, 2024, immediately after hyaluronidase administration. Marked erythema and swelling covering an area of approximately 20 × 10 cm. The skin is tense and slightly shiny, indicating the onset of a mild acute inflammatory reaction following paclitaxel extravasation. No open wounds, blistering or bleeding present. Surrounding skin shows normal vascularity without signs of ischemia. Without immediate changes after hyaluronidase administration. **(A)** Right antecubital fossa, anteromedial view: erythema and swelling surrounding the venipuncture site; small superficial vesicle with mild ecchymosis at injection sites. **(B)** Right upper limb, anterolateral view from a proximal angle showing circumferential extension of erythema/induration without blistering or skin breakdown. Imaging details: ambient lighting, no flash, no digital enhancement, minor cropping; no scale bar.

A 63-year-old female patient with a metastatic malignant tumor of the endocervix was receiving palliative chemotherapy consisting of Kolliphor EL-formulated paclitaxel and carboplatin administered every three weeks via a 20-gauge peripheral catheter. At the beginning of the paclitaxel infusion (330 mg in 500 mL normal saline) during the third cycle, the patient reported a mild pressure at the infusion site. Physical examination revealed a large edema, approximately 20 x 10 cm in size, located in the right cubital region, involving the entire circumference of the upper arm (**Figure 1A, B**).

The infusion was immediately stopped, and the attending nurse attempted to aspirate the extravasate through the catheter but yielded no return. Based on visual inspection of residual volume in the IV container and estimated time of extravasation from the start and the rate of infusion, the medical team estimated that approximately 50–100 mL of paclitaxel leaked to the affected site.

Subsequently, patient management was initiated according to our institutional protocol (summarized in **Table 1**) with hyaluronidase and the application of dry warm compresses with “disperse and dilute” intent. First, prior to the subcutaneous injections and immediately before cannula withdrawal, a volume of 0.4 mL (60 IU) of hyaluronidase solution was delivered through the cannula lumen to facilitate dispersion at the presumed site of highest paclitaxel concentration. Then, following the ESMO–EONS (10) guidelines, we delivered multiple small perilesional subcutaneous injections with a total dose of hyaluronidase within recommended 150–900 IU range. The patient reported only minor pain related to the injections, and no discomfort attributable to either extravasation or hyaluronidase; therefore, analgesics were not required.

**Table 1:** Institutional algorithm for cytostatic drug extravasation management.

Step 1: Stop infusion immediately, leave cannula in place.			
Step 2: Carefully try to aspirate drug, then remove cannula. If hyaluronidase is to be given, leave cannula in place.			
Step 3: Inform head nurse, pharmacy and physician. Document type and dose of leaked drug.			
Step 4: Apply antidote (if the antidote is unavailable - skip to step 5)			
Vinca alkaloids and taxanes: Subcutaneous application of hyaluronidase within one hour: inject hyaluronidase diluted to 150 IU/mL perilesionally with multiple injections of 0.1 - 0.2 mL volume, 0.4 mL may be administered directly via cannula. Dose individualized at the physician’s discretion.	Anthracyclines, antibiotics, alkylating agents: Local application of 99% DMSO within 10 minutes. Apply thin layer of topical DMSO using gauze on dry skin and allow to dry. Do not apply excessive pressure.		All other drugs: Skip to step 5
Step 5: Apply thermal therapy.			
Vinca alkaloids, taxanes, oxaliplatin: Dry heat for 20 minutes, four times daily for one to two days after the extravasation.		All other drugs: Dry cold for 20 minutes, four times daily for one to two days after the extravasation.	
Step 6: Elevate the limb.			
Step 7: Patient education on home care and regular monitoring of the affected site.			

DMSO- dimethyl sulfoxide

**Figure 2:**
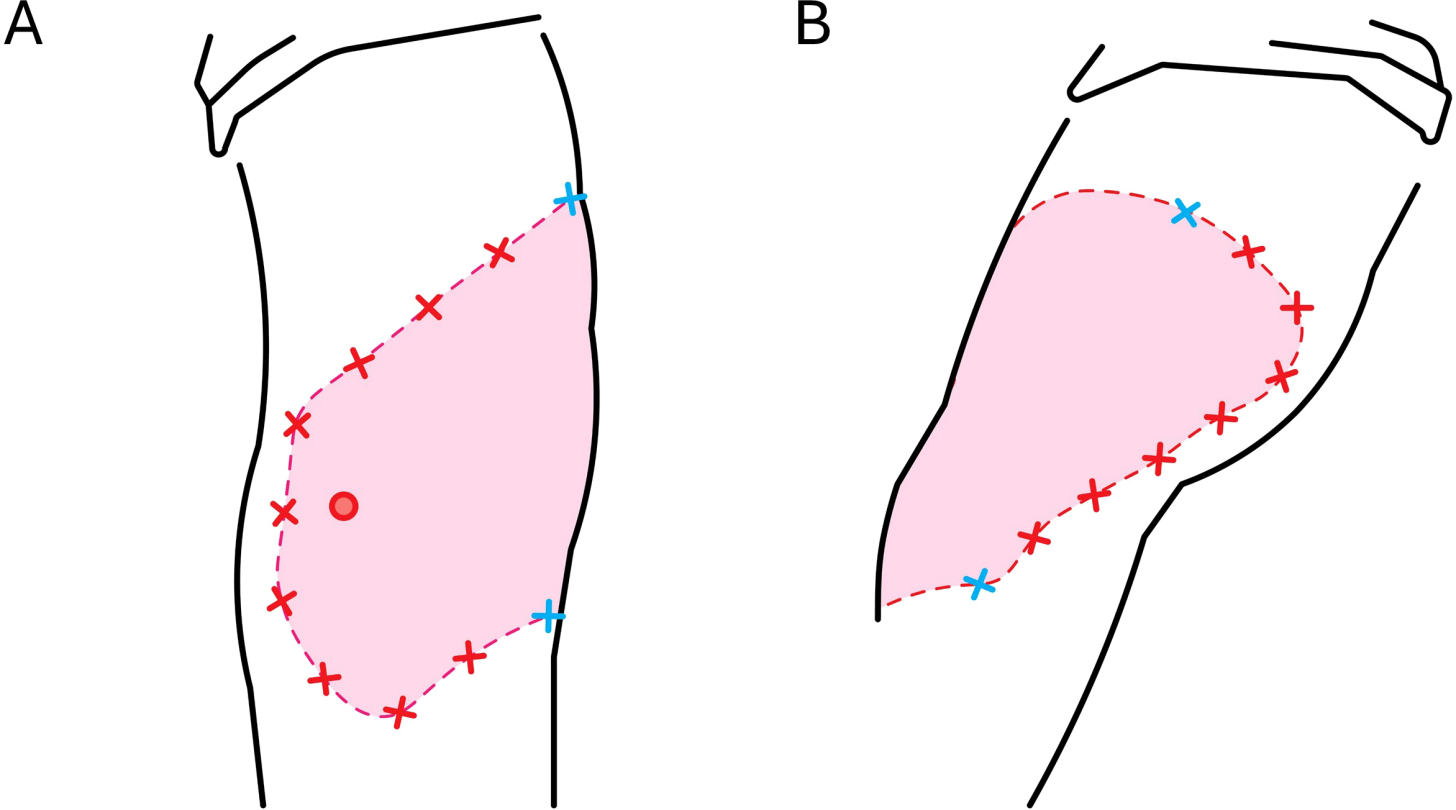
Schematic diagrams illustrate the locations of the hyaluronidase administration around the extravasation site. Hyaluronidase was prepared aseptically in the adjacent hospital pharmacy cleanroom under laminar airflow hood. A pharmacist diluted preservative free hyaluronidase (Hylase^®^Dessau150 I.E., Esteve Pharmaceuticals GmbH, batch/lot 2960819) according to the manufacturer’s instructions with 1 mL normal saline to the concentration of 150 IU/mL and prefilled 18 single use 1 mL syringes with 0.2 mL each, and one syringe with 0.4 mL to ensure accurate dosing. All syringes were capped with 25-G single use needle to avoid needle dulling and were immediately transported to the administration site. Before injection, the extravasation field was cleansed with a surgical skin antiseptic over a broad area (applied with sterile gauze and allowed to dry). Red crosses indicate the approximate locations of the 18 subcutaneous injections: 0.2 mL (30 IU) of hyaluronidase was injected at each site in an evenly spaced pattern around the visibly erythematous and edematous field, with the needle inserted at ~45° to a depth of ~1 cm. Blue crosses mark common reference points visible from both angles to indicate anatomical correspondence between the anteromedial **(A)** and anterolateral **(B)** views. The red circle denotes the peripheral intravenous cannula site. Immediately before cannula removal, 0.4 mL (60 IU) of the same solution was instilled slowly through the cannula lumen to promote dispersion at the presumed site of highest paclitaxel concentration. Hyaluronidase was administered within one hour after extravasation recognition, without local anesthesia.

A total number of 18 subcutaneous injections of 0.2 mL (30 IU each) were administered evenly around the erythematous area within one hour after the extravasation was identified. A schematic diagram with full technical details on preparation, dosing and needle placement is presented in **Figures 2A, B**. The number of injections was determined by lesion size rather than estimated leakage volume. Injection-related effects were limited to mild capillary bleeding and small ecchymoses, as shown in **Figures 1A, B** (and faintly in **3B**).

Following the administration, dry warm compresses in the form of hot packs were applied to the extravasation site for 20 minutes. The medical team consisting of oncologist, oncology nurse, and pharmacist performed pain monitoring, as well as serial reassessments of color changes, erythema/induration, local temperature, and bleeding at injection sites.

The remaining chemotherapy was administered via a peripheral catheter inserted in the contralateral arm. The patient was subsequently discharged with verbal and written instructions to apply dry warmth to the affected area for 20 minutes, four times daily for the next two days and to monitor the site for any signs of progression or complications: specifically pain, erythema, fever or functional decline.

**Figure 3:**
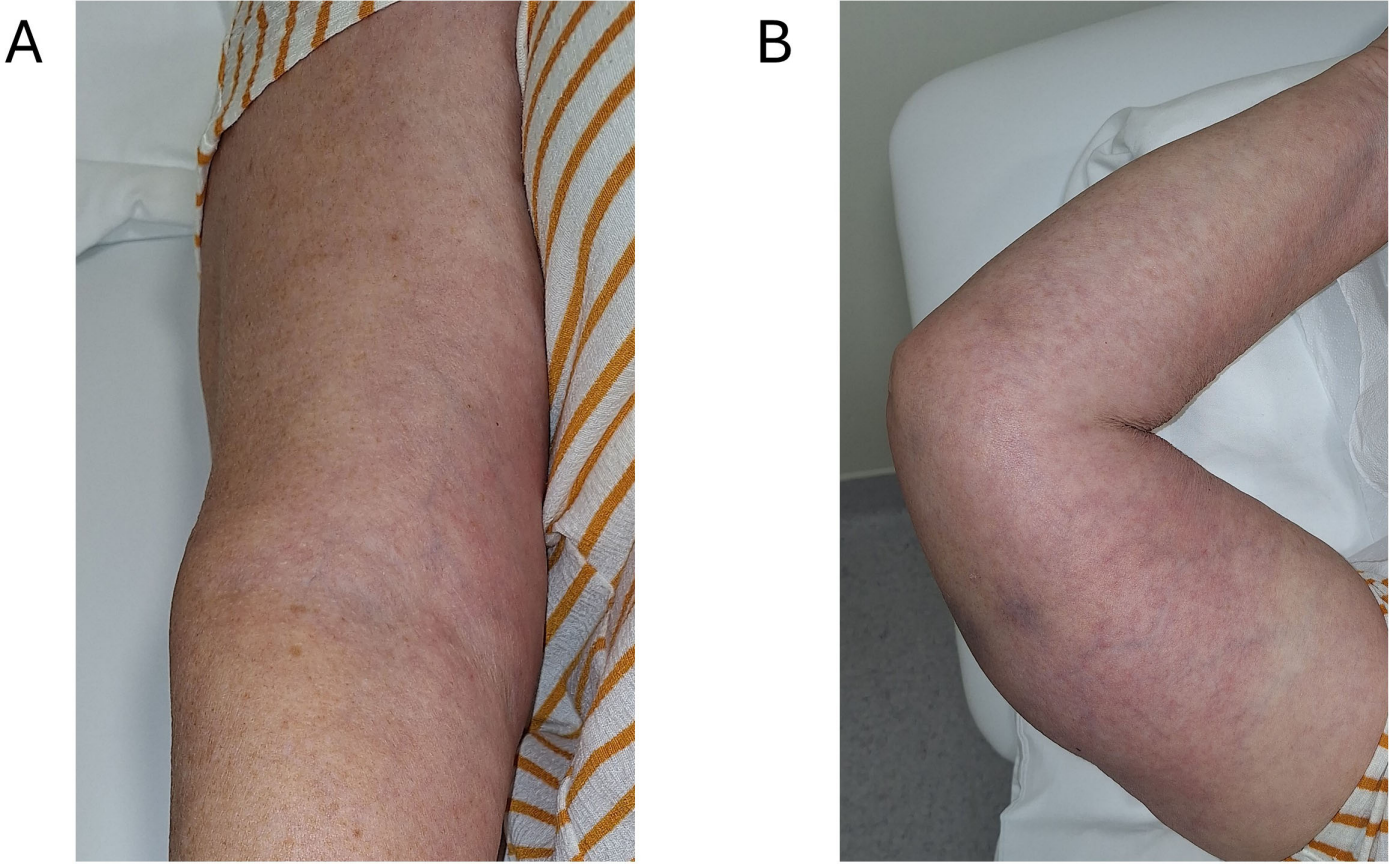
Early clinical improvement. September 13^th^, 2024. Mild erythema and bruising with intact skin, no necrosis, swelling or blistering, indicating healing four days after extravasation. **(A)** Right antecubital fossa, anteromedial view similar to **Figure 1A**. Erythema intensity visibly reduced; induration softening; no bullae or ulceration. **(B)** Right upper limb, anterolateral view similar to **Figure 1B**. Residual pink discoloration with resolving ecchymosis at prior injection sites; skin remains intact. Imaging details: ambient lighting, no flash, no digital enhancement, minor cropping; no scale bar.

During follow up, oncology assessments on days 2 and 4 showed stable or improving lesion margins, absence of blistering, and maintenance of an afebrile status. On day 2, the patient was scheduled for insertion of a peripherally inserted central catheter (PICC) into the left arm to enable continuation of treatment. The PICC was placed under ultrasound and ECG guidance, with chest radiograph confirming the tip position approximately 30 mm below the carina (procedure completed on 30 September 2024). Although central access reduces, but does not eliminate the risk of extravasation (10, 11), our institution did not mandate central access for taxanes administration at the given time, and public healthcare reimbursement limited routine PICC placement. Following the presented event, internal education was reinforced, and PICC use is now encouraged for high-risk agents such as paclitaxel. The affected skin on day 4 is presented in **Figures 3A, B**.

Thereafter, nine days after the event, the patient was evaluated by a dermatologist due to persistent itching at the affected area. Dermatological examination revealed residual pink-red erythema on the palmar side of the forearm and the medial upper arm. The affected skin was warmer to the touch but without any palpable induration or fluctuance, and no residual edema was observed.

Duplex ultrasonography of the right upper limb showed normal flow in the deep veins and arteries within the examined region. There was no evidence of thrombosis or luminal occlusion in the deep venous system at that time. The dermatologist concluded that the findings were consistent with irritant dermatitis resulting from paclitaxel extravasation.

The patient was prescribed levocetirizine 5 mg for use in the evening as needed for itching, and topical betamethasone cream (0.5 mg/g) to be applied initially twice daily for three days, followed by once daily administration in the evening for anti-inflammatory control of erythema and pruritus. A petrolatum (white soft paraffin) emollient was prescribed to reduce transepidermal water loss and to support barrier repair. Pruritus improved promptly within several days. Systemic or topical antibiotics were not indicated, as no clinical signs of infection were present. The patient was instructed to seek immediate evaluation in case of any deterioration, particularly fever or chills.

### 2.2 Subsequent phase

Subsequently, patient received the fourth cycle of chemotherapy. Pre-administration assessment confirmed clinically stable extravasation field (intact skin, no blistering), no photographs were taken at this visit as there were no clinically meaningful local findings to document, and chemotherapy proceeded via the PICC uneventfully. Two weeks later, during the follow-up visit, patient complained of general weakness, fatigue, difficulty sleeping and pronounced paresthesia in the right upper limb. Notably, there were no signs of bleeding, erythema or swelling at the site of previous extravasation. Laboratory tests revealed grade 3 leukopenia, thrombocytopenia and anemia, and grade 4 neutropenia without fevers. As a result, the patient received pegfilgrastim and blood transfusions. The following cycle of chemotherapy was postponed until hematologic recovery was achieved.

Within three weeks post-extravasation, the patient developed persistent neuropathic symptoms localized to the right upper limb, characterized by pain and sensory deficits in the ulnar nerve distribution, specifically involving the fourth and fifth digits, without any motor impairment. It is noteworthy that paresthesia in both upper and lower limbs, as well as a decline in hematologic recovery, worsened following the subsequent cycle of chemotherapy administered after extravasation.

One month after the case, a comprehensive clinical neurology examination identified sensory neuropathy with ulnar-territory hypesthesia in the affected limb and distal length-dependent changes consistent with paclitaxel-induced peripheral neuropathy (PIPN). No motor or other alarming features were detected, and the findings were sufficiently characteristic to preclude the need for additional investigations such as nerve conduction studies, electromyography, or neuroimaging. Pregabalin 75 mg was initiated once daily at bedtime (accompanied by oral vitamin B6–300 mg once daily) then twice daily, with symptomatic improvement. The overall chronology and examination findings support a dual etiology: mild focal ulnar-territory irritation secondary to extravasation, superimposed on early PIPN from systemic therapy. A detailed timeline of the patient’s symptoms, clinical findings, and interventions is summarized in **Table 2**.

**Table 2:** Timeline of the clinical course.

Date	Event	Management
09-Sep-2024	Paclitaxel (330 mg in 500 mL NS) extravasation during third cycle of chemotherapy (approx. 50–100 mL); marked erythema and swelling on the right arm.	Hyaluronidase 150 IU/mL: 0.4 mL intraluminal via cannula (60 IU) + 18 × 0.2 mL SC perilesional (30 IU each; 540 IU). Total 600 IU administered within one hour after recognition. Dry warm compresses 20 min., four times a day for ~48 h. Time stamped photo taken (**Figure 1**). Rest of chemotherapy (paclitaxel + carboplatin 560 mg) administered via peripheral catheter to contralateral arm.
11-Sep-2024	Oncology examination: visible improvement of local symptoms, no blistering, afebrile.	PICC scheduled to reduce further peripheral risk during therapy.
13-Sep-2024	Oncology examination: Further visible improvement of local symptoms, no blistering, afebrile.	Time stamped photo taken (**Figure 3**).
18-Sep-2024	Dermatology examination: Persistent itching and residual pink-red erythema at extravasated site. Skin warmer, without induration, fluctuance, edema. Duplex ultrasonography: normal flow in the deep veins and arteries, no evidence of thrombosis or luminal occlusion. Diagnosis: irritant dermatitis after paclitaxel extravasation.	Prescribed levocetirizine 5 mg for use in the evening PRN for itching, topical betamethasone cream (0.5 mg/g) twice daily for 3 days, once daily thereafter. Emollient for skin hydration and barrier protection.
Following days	Self-reported improvement of erythema and pruritus.	–
30-Sep-2024	PICC insertion to the left arm.	PICC inserted under USG/ECG guidance, tip ~30 mm below carina.
01-Oct-2024	Oncology examination before fourth cycle of chemotherapy: extravasation field stable, intact skin, no blistering.	Paclitaxel 330 mg and carboplatin 560 mg via PICC uneventful.
15-Oct-2024	Neurology consult: sensory neuropathy with ulnar-territory hypesthesia in extravasation limb (local irritation) + length-dependent distal sensory changes in fingers/toes compatible with PIPN; no motor deficit/red flags. Labs: grade 3 leukopenia, thrombocytopenia, anemia; grade 4 neutropenia. ADL: difficulty sleeping due to neuropathic pain.	Neurology: Prescribed pregabalin 75 mg (0–0–1), vitamin B6–300 mg (1–0–0). Oncology: pegfilgrastim + blood transfusions given.
28-Oct-2024	Neurology/oncology: symptomatic improvement of neuropathy on pregabalin, paclitaxel discontinued ADL: improved sleep.	Neurology: Pregabalin ↑ 75 mg (1-0-1). Carboplatin 560 mg monotherapy via PICC uneventful.
18-Nov-2024	Neurology: further improvement of neuropathy.	Conservative management maintained.
Following weeks	Gradual improvement of local and systemic symptoms.	
28-Jul-2025	Ten-month follow-up: local soft-tissue symptoms resolved. Neurological exam normal except for mild persistent paresthesia. Intermittent pruritus, no permanent damage from extravasation.	Pharmacist recommended topical dimetindene gel (1 mg/g) PRN.
Following weeks	Pruritus relief with dimetindene.	

ADL- activities of daily living; ECG- electrocardiogram; NS- normal saline; PICC- peripherally inserted central catheter; PIPN- paclitaxel-induced peripheral neuropathy; PRN- as needed; SC- subcutaneously; USG- ultrasonography

Due to the combined local and systemic adverse effects of paclitaxel, treatment was continued with carboplatin monotherapy. Regarding the extravasation, local soft tissue symptoms have resolved completely. Neuropathic complications have also improved to the extent that systemic treatment is no longer required. However, the patient continued to experience occasional itching at the site of extravasation. At the 10-month follow-up, the oncology pharmacist recommended topical dimetindene gel (1 mg/g) as needed for pruritus (weeks later, patient reported relief when using the gel). Paresthetic sensations persisted in the fingers of both hands, with slightly greater intensity on the side of the extravasation, though neurological examination remains within normal limits. At the 10-month follow-up, no further permanent damage attributable to extravasation was observed.

Throughout the entire care and patient management, institutional ethical rules were strictly followed. Also, signed patient consent for publication of the case was obtained during the follow-up.

## 3 Discussion

In this case report, we described successful management of high-volume paclitaxel extravasation by concomitant application of hyaluronidase and dry warmth. Given the large volume of concentrated paclitaxel involved, this approach was considered most appropriate to facilitate drug dispersion and dilution and to minimize local tissue injury.

Paclitaxel is a widely used chemotherapeutic agent, indicated for a variety of malignancies, including cervical cancer. Conventional paclitaxel is formulated with Kolliphor EL, a biologically active surfactant associated with systemic hypersensitivity reactions. No comparative clinical data suggest that this vehicle exacerbates extravasation severity compared with albumin-bound paclitaxel, and current guidelines do not differentiate management based on formulation excipients (10, 11, 13). Also, as outlined in the Introduction, paclitaxel extravasation is scarcely reported, which likely reflects the increasing use of central venous catheters that may reduce the risk of extravasation (1, 11). When the drug leaks into surrounding tissue, it can lead to local tissue injury ranging from mild inflammation to severe necrosis. Symptoms can be immediate or delayed, sometimes appearing several days or weeks after the incident (4).

Since there is little experience with this adverse drug event, also its management is less well known. To date, only one study has directly compared two treatment approaches. Du Bois et al. (9) presented their experience with paclitaxel extravasated in four patients; two patients were treated with hyaluronidase and cold packs, while the other two received cold packs alone. Hyaluronidase is an enzyme able to degrade hyaluronic acid in the interstitial space, thereby increasing tissue permeability and promoting dispersion of the extravasated drug. Local reactions including swelling, mild pain, erythema, induration, and hyperpigmentation were observed in all patients; however, no ulceration. All local reactions were resolved within two weeks, but the two patients treated with hyaluronidase experienced a longer duration of symptoms. The authors concluded that paclitaxel extravasation generally induces only mild soft tissue reactions and recommended cooling as the standard treatment rather than hyaluronidase. This recommendation aligns with the JSCN–JSMO–JASPO guidelines (1).

On the contrary, the 2012 ESMO–EONS guidelines recommend the use of hyaluronidase in combination with dry warm compresses for the management of taxane extravasation (10). Dry warm compresses promote vasodilatation and facilitate the absorption of extravasated drug from tissue sites. This approach is generally recommended for management of non-DNA binding vesicants. However, evidence supporting its efficacy in the treatment of paclitaxel extravasation is limited in real-world practice, especially compared to its established use in vinca alkaloids (another group of non-DNA-binding vesicants) (1, 10). The ESMO–EONS recommendation may therefore be extrapolated from clinical experience with vinca alkaloid extravasations. Both most recent guidelines (1, 11) explicitly acknowledge the very limited evidence base regarding the optimal management of this complication. However, their recommendations diverge. JSCN–JSMO–JASPO advise against the use of warmth and hyaluronidase for taxanes and issue a weak recommendation for cold compresses to limit progression. In contrast, the ONS–ASCO guidelines conditionally recommend the use of an antidote (hyaluronidase) in taxane extravasation (including paclitaxel) and suggest warm compresses as an acceptable thermal intervention, while emphasizing the very low certainty of evidence. Here, we have successfully used the variant with warm compresses.

The choice between cold and warm compresses in paclitaxel extravasation remains debatable, partly due to the drugs’ neurotoxic potential. Notably, limb cryotherapy is increasingly employed to mitigate the paclitaxel-induced peripheral neuropathy (14). A recent systematic review and meta-analysis reported an association between distal-extremity cooling during paclitaxel administration and a reduced incidence of peripheral neuropathy, although the certainty of evidence varied (15). From a mechanistic standpoint, warmth-induced vasodilatation could theoretically exacerbate neuropathic risk. In our case, however, we prioritized a “disperse and dilute” strategy to minimize the likelihood of local tissue injury, considering this risk to outweigh the theoretical neuropathy concern. Additional support for the use of warm compresses in paclitaxel extravasation is provided by a recently published case report (5), which described a favorable outcome despite delayed recognition and treatment. In that case, the patient developed an indurated, erythematous plaque on medial upper arm and forearm blisters five days after extravasation. Management included three consecutive daily intravenous boluses of high-dose methylprednisolone, followed by oral prednisone, systemic antibiotics, and warm compresses. Symptoms improved within the first week, and the lesion resolved over the following three weeks. Although this case differed from ours with respect to timing and the use of systemic corticosteroids and antibiotics, its favorable course further supports the potential benefit of warmth in facilitating the resolution of taxane-related extravasation inflammation. In contrast, our case involved immediate recognition and early administration of hyaluronidase with dry warmth, resulting in rapid and complete cutaneous recovery without the need for systemic anti-inflammatory therapy. This experience also emphasizes the benefit of established local guidelines in ensuring rapid, coordinated management of adverse events.

To the best of our knowledge, no previously published real-world data, case reports, case series, or reviews have described paclitaxel extravasation managed with this approach. A targeted narrative literature review (PubMed and Google Scholar) using the terms “paclitaxel extravasation,” “taxane extravasation,” “hyaluronidase,” and “warm compress(es)” identified only one partially comparable report by Du Bois et al. (9), in which hyaluronidase was combined with cooling rather than warmth. In contrast to the prolonged recovery reported by Du Bois et al., our patient achieved rapid resolution of erythema and swelling without ulceration or induration, despite a higher estimated extravasated volume. This case contributes to the limited body of evidence by documenting a favorable clinical outcome following prompt administration of hyaluronidase with dry warmth.

Despite successful local management, the patient experienced persistent paresthesia in the affected limb, which may reflect a combination of paclitaxel-induced peripheral neuropathy and local nerve irritation from the leakage. Notably, paresthesia was also reported in the lower limbs and was present after the first chemotherapy cycle, suggesting a systemic neurotoxic effect of paclitaxel. Paclitaxel-induced peripheral neuropathy is a well-recognized dose-limiting toxicity of taxanes and often requires modifications to treatment regimen or its discontinuation (14), as occurred in the presented case. Paclitaxel induces neurotoxicity primarily by disrupting microtubule dynamics in distal sensory axons, impairing their ability to remodel and adapt, which leads to neurite retraction and degeneration predominantly affecting sensory function. These effects have been observed after local exposure of nerves *in vitro* (16).

A similar case reported neuropathic complications following paclitaxel extravasation where the patient developed burning pain, sensory loss and motor weakness localized to the affected limb several days after infusion (12). In that case, no specific measures were taken, suggesting that spreading of the extravasated paclitaxel in our approach did not lead to additional harm. These findings imply that the observed neuropathic complications may have been unavoidable due to the neurotoxic nature of paclitaxel. However, it remains unclear whether our intervention prevented more severe neuropathic outcomes. Given current clinical recommendations, conducting a study without any local management would be considered unethical.

This report has certain limitations. Standardized photographic or metric documentation was not performed. The absence of validated skin-injury scoring limits objectivity of local outcome reporting, though clinical healing was complete and uncomplicated. Another key limitation is the inability to definitively distinguish neuropathic symptoms caused by extravasation from those resulting from systemic chemotherapy-induced neurotoxicity. It is also possible that the continued administration of paclitaxel after the extravasation event contributed to the worsening of symptoms, and that earlier discontinuation of the drug might have mitigated further neurological damage.

## 4 Conclusion

The combination of hyaluronidase and dry warmth appears to be a viable option for preventing tissue damage following high-volume paclitaxel extravasation. However, this approach may not prevent the development of neuropathic complications, which are likely attributable to the neurotoxic nature of paclitaxel. This case also underscores the importance of administering vesicant chemotherapy agents via central venous catheters rather than peripheral lines, as the risk of extravasation is significantly reduced. Further research is needed to establish standardized management protocols and to evaluate the comparative effectiveness of different interventions for paclitaxel extravasation.

## 5 Patient perspective

The patient appreciated the prompt management of extravasation, having been informed beforehand and concerned about the potential skin damage caused by extravasated paclitaxel. Patient reported no additional pain or discomfort attributable to the hyaluronidase itself, aside from the brief discomfort of the needle injections. Subjectively, patient stated that the warm compresses provided the greatest relief of pain and other symptoms during initial days following extravasation. Patient continued to occasionally apply warmth to the affected site for approximately two weeks after the incident, finding it soothing. Patient received verbal and written home-care instructions after the incident and was encouraged to report any new or worsening symptoms related to the extravasation. During the first week, she predominantly experienced itching rather than pain at the site and reported no limitations in activities of daily living attributable to the local injury. Acute pruritus improved promptly after initiation of betamethasone cream and levocetirizine. After the fourth cycle of chemotherapy, she reported difficulty sleeping due to discomfort from paclitaxel-induced peripheral neuropathy; sleep improved within two weeks following initiation of pregabalin 75 mg. The patient later reported occasional mild itching at the former extravasation site and consulted the oncology pharmacist; topical dimetindene gel provided meaningful relief.

## Data Availability

The original contributions presented in the study are included in the article/supplementary material. Further inquiries can be directed to the corresponding author.
